# MicroRNA-126 engineered muscle-derived stem cells attenuates cavernosa injury-induced erectile dysfunction in rats

**DOI:** 10.18632/aging.203057

**Published:** 2021-05-23

**Authors:** Zihao Zou, Muyuan Chai, Feixiang Guo, Xin Fu, Yu Lan, Shuqi Cao, Jianan Liu, Long Tian, Geng An

**Affiliations:** 1 Center for Reproductive Medicine, Key Laboratory for Major Obstetric Diseases of Guangdong Province, Third Affiliated Hospital of Guangzhou Medical University, Guangzhou, Guangdong, PR China; 2 National Engineering Research Center for Tissue Restoration and Reconstruction, South China University of Technology, Guangzhou, Guangdong, PR China; 3 Beijing Chao-Yang Hospital, Capital Medical University, Beijing, PR China

**Keywords:** erectile dysfunction, cavernosa injury, muscle-derived stem cells, microRNA-126, exosomes

## Abstract

Background: Cavernosa injury is a common cause of organic erectile dysfunction (ED), which requires safe and effective treatments. In the present study, the therapeutic efficiency of muscle-derived stem cells (MDSCs) modified with microRNA-126 (miR-126) was determined in rats with cavernosa injury.

Methods: MDSCs were transfected with miR-126 and then were transplanted into rats with cavernosa injury. Erectile function, vascular function (western blot and immunofluorescence), extraction, and detection of exosomes were then undertaken.

Results: On the 28th day after transplantation, the highest value of intra-cavernous pressure (ICP)/mean arterial pressure (MAP) in rats of miRNA-126 group (0.84 ± 0.14) was observed (Control: 0.38 ± 0.07; MDSC: 0.54 ± 0.11, Vector: 0.60 ± 0.02; respectively). Treatment of miRNA-126-modified-MDSCs remarkably strengthened vascular structure, supported by hematoxylin-eosin staining. The expression of CD31, von Willebrand Factor and vascular endothelial factors were higher than those in other groups, indicating improved vascular function. *In vitro* mechanism studies showed that exosomes containing miR-126 isolated from MDSCs promoted angiogenesis and attenuated apoptosis of human umbilical venous endothelial cells. Finally, insulin receptor substrate 1 and Krüppel-like factor 10 were determined as the direct target genes of miR-126.

Conclusions: MiR-126 engineered MDSCs notably repaired cavernosa injury in rats via vascular reconstruction by directly targeting IRS1 and KLF10, in which the exosomes secreted by MDSCs played a critical role.

## INTRODUCTION

Erectile dysfunction (ED) is a common sexual dysfunction disease in male sexual maturity, which can be divided into organic ED, spiritual ED, psychological ED and secretory ED according to its pathogenesis [1, 2]. It has been reported that in patients with organic ED, injured cavernosa-induced ED predominated [2]. Corpus cavernosum is a vascular-rich tissue [3]. Healthy cavernosum tissues are essential for maintaining normal erectile function, which are depended on the circulation and blood supply in the cavernous sinus [3]. Thus, the reconstruction of the microvascular system plays a vital role in the improvement of ED induced by cavernosa injury. Existing treatments, such as allopenis transplantation, acellular matrix replacement, and local injection of vasoactive drugs into the corpora cavernosa, have poor efficacy and are accompanied by many adverse reactions [4, 5]. Therefore, safer and more effective treatments for cavernosa injury are worth investigated.

Muscle-derived stem cells (MDSCs) belong to adult pluripotent stem cells, which have the ability of multicellular differentiation and self-renewal [6]. Recently, studies have indicated that MDSCs are involved in ED treatment. Nolazco et al. [7] found that MDSCs implanted in the corpus cavernosum can differentiate into smooth muscle cells (SMCs), and the expression of α-SMA and endothelial nitric oxide synthases significantly were increased. Kim et al. [8] demonstrated that LAZC-transfected-MDSCs injected into rats with cavernosa injury increase intracavernous pressure (ICP) and the production of PGP 9.5. Moreover, our previous study indicated that corpus cavernous seeded with MDSCs improve the function of injured cavernosa [9]. However, fibrosis and necrosis are found in the inoculation area due to a lower capillary formation [9]. Therefore, we committed to promote the formation of vascular tissue after MDSCs plantation for the repair of cavernosa injury.

MicroRNAs has been reported to mediate differentiation and self-renewal of stem cells [10]. MiR-126 is highly expressed in endothelial cells (ECs) [11]. It promotes the metastasis of ECs and capillary formation, and prolongs cell growth cycle.11 Knockout of miR-126 results in vascular integrity damage and hemorrhages [12]. Moreover, some studies indicated the essential role of microRNA-126 (miR-126) in the regulation of smooth muscle growth, differentiation, and function [13–15]. Zhou et al. [13] suggested that endothelial miR-126 is a key intercellular mediator to elevate the contents of SMCs. Santulli et al. [14] indicated the specific roles of miR-126 in the regulation of ECs and VSMC function. Additionally, Zhu et al. [15] demonstrated that miR-126 in adipose tissue-derived stem cells (ADSCs)-derived exosomes can help to enhance the contents of EC and smooth muscle in diabetic rats.

In this study, miR-126 was transfected into MDSCs for the establishment of miR126-overexpressing-MDSCs, which then were injected into rats with cavernosa injury. Their effects on vascular reconstruction of cavernosa injury and the underlying mechanisms were investigated.

## METHODS

### Overexpression of miR-126 in MDSCs

MDSCs from primary rats were isolated from rat muscle tissues, and cells were grown in Dulbecco's modified Eagle medium with Low glucose (DMEM [L], Gibco, NY, USA) containing 10% fetal bovine serum (FBS, Gibco) and 1% penicillin–streptomycin (Gibco). The concentration of CO2 and temperature in the incubator were kept at 5% and 37°C respectively. After 3–5 generations, cells with about 80% fusion were used for the subsequent experiments.

Lentivirus expressing microRNA-126 (lentivirus-microRNA-126-GFP) and GFP (lentivirus-vector-GFP) were provided by Thermo Fisher Scientific (Waltham, MA, USA). MDSCs were transfected according to the instructions after they were transferred into the 12-well plates (1 × 10^4^/well). After treatment overnight, MDSCs were trypsinized, centrifuged, and maintained in DMEM [L] medium adding lentivirus-microRNA-126-GFP or lentivirus-vector-GFP. After 12 hours, immunofluorescence was performed for the identification of transfection efficiency. Then, MDSCs continuously were grown for another 3–5 days.

### Cell viability assay

After transfection, MDSCs were cultured in 96-well plates (5 × 10^3^ cells in 100 mL/well). According to the instructions of Cell Counting Kit 8 (CCK8, Beyotime, Shanghai, China), cells were added with solution (10 μL/well) and then cultured for 1 h. A versatile microplate reader (Leica, Wetzlar, Germany) was used for the measurement of optical (OD) density value at 450 nm.

### Animal experiments

Male Sprague-Dawley rats (*n* = 40) were randomly divided into 4 groups: Control, MDSCs, Vector, and miRNA-126. The specific protocols were as follows: After anaesthetization with 1% pentobarbital sodium (Beyotime), rats were cut off a median incision of about 1 cm above the penis. In the middle of penile dorsal, a gap of about 0.2 cm long and 0.1 cm deep was cut in the left or right corpus cavernosum [16]. While the incision was closed, rats in the Control group were injected with 200 μL phosphate balanced saline (PBS); rats in the MDSC group were transplanted with MDSCs (1 × 10^6^ in 200 μL PBS) via the intracorporal injection into the corpus cavernosum of penis; rats in the Vector group were transplanted with MDSCs which transfected with blank vector (1 × 10^6^ in 200 μL PBS); rats in the miRNA-126 group were transplanted with MDSCs transfected with miR-126 (1 × 10^6^ in 200 μL PBS).

### Measurement of erectile function

On the 14th and 28th day after the operation, the erectile function of rats was tested. After the anaesthetization, rats were cut along the midline of the neck and abdomen until the carotid artery and the cavernous nerve were exposed. Then, a heparin saline-filled catheter was inserted into the artery and used for the record of mean pressure. Bipolar electrode was used for the stimulation of nerves, which was connected to pressure sensor to PowerLab physiological recorder (AD Instruments, Australia). ICP and mean arterial pressure (MAP) were recorded. After the treatment, rats were euthanized, and the penile tissues were removed for following experiments.

### Real-time quantitative reverse transcriptase (qRT-PCR)

Trizol Reagent Kit (Thermo Fisher Scientific) was used for extraction of total RNA from MDSCs and rats. The concentrations of total RNA were determined by a Spectrophotometer (Nanodrop 2000, Thermo Fisher Scientific). Then, PrimeScript RT reagent kit (Takara, Shiga, Japan) was performed for the reverse transcription of RNA into cDNA, which was used as template in the following amplification experiment by using the SYBR Premix ExTaqII (TliRNaseHPlus) Kit (Takara). Quantitative levels of genes were controlled by β-actin. All primers used are listed in Supplementary Table 1.

### Western blot analysis

BCA protein assay kit was obtained from Beyotime, and was used for the isolation of total proteins from MDSCs and rat tissues. After quantification, extracted proteins were loaded on 8–12% SDS-PAGE and separated for 60–100 min. Then, proteins were transferred to PVDF membrane on ice for 120–150 min. After that, protein-loaded membranes were blocked in 5% BSA at room temperature for 1–1.5 h, and then treated with primary antibodies against IRS1 (Cell Signaling Technology, CST, Cat.#2382), KLF10 (Abcam, Cat.#ab184182), α-SMA (CST, Cat.#19245), CD31 (CST, Cat.#77699), vWF (CST, Cat.#65707) and VEGF (CST, Cat.#2463) overnight at 4°C. After incubating with relative secondary antibody at room temperature for 1 h, the images of bands were acquired by ECL quantification detection. All antibodies used above were purchased form Cell Signaling Technology (Beverly, MA, USA) and Santa Cruz Biotechnology (Santa Cruz, CA, USA).

### Cell culture

Human umbilical venous endothelial cells (HUVECs) were obtained from the Cell Bank of Chinese Academy of Sciences (Shanghai, China). After resuscitation, cells were grown in ECM medium containing 5% fetal bovine serum. Then, the medium was replaced every 2 days. After cells grown to 80–90% monolayer, they were subcultured for further experiments.

### EXs isolation from transfected MDSCs

After transfection as described above, MDSCs were cultured for another 72 h. Then, cell medium was collected and used for the isolation of EXs with differential centrifugation. First, cells were collected and centrifuged at 300 × g and 2000 × g for 10 minutes, respectively, to remove the cells and apoptotic fragments. Then collected sediment was centrifuged at 10000 × g for 30 minutes to remove the vacuoles. Finally, after the centrifugation at 4°C for 90 minutes at 100000 × g, the remaining sediment was resuspended by PBS, and then centrifuged at 100000 × g for another 90 minutes. The extracted EXs was identified by transmission electron microscopy (TEM).

### EXs labeling and internalization

After labeling with PKH67 fluorescent dye (Sigma) for 30 min at room temperature, 10 μg/mL MDSCs-derived EXs or control PBS were co-cultured with HUVECs for 24 h. Then 4% paraformaldehyde was used for fixing. After washing with PBS, HUVECs with labeled-EXs were stained with DAPI (1:500, Invitrogen) for 5 min at room temperature. The respective images were captured by a Zeiss LSM 780 confocal microscope.

### Transwell migration assays

HUVECs were cultured in a 24-well plate with 70% density. Matrigel (BD Biosciences, San Jose, CA, USA) was placed on the Transwell chamber membrane and placed overnight in the cell incubator at 37°C. After being resuspended with serum-free medium, 100 μL cells (5 × 10^5^/Ml) were added into the Transwell chamber containing 500 μL medium. After 36 h of culture, the medium was discarded, and then 500–700 μL 0.1% crystal violet staining was added for staining for 15 min at room temperature. After dyeing, cells were washed with PBS for 3 times. Finally, cells were counted under a microscope in different fields.

### Luciferase reporter assays

According to the instructions, IRS1 or KLF10 3′UTR sequences were amplified by PCR and inserted into pMIR vector. 1 × 10^4^ MDSCs were cultured in 96-well plates and co-transfected with pMIR-reporter and pMIR-IRS1 or pMIR-KLF10 plasmids with miR-126 mimic/ inhibitor by Lipofectamine 2000 (Invitrogen, USA) for 24 h. Luciferase activity was measured and analyzed by a multifunctional fluorescent enzyme marker (Tecan, Switzerland).

### Immunofluorescence staining

MDSCs and slices of rats were fixed using 4% paraformaldehyde (Servicebio, Wuhan, China) and permeabilized using 0.05% TritonX-100 (Servicebio). After blocking, MDSCs and slices of rats were stained with primary antibodies against α-SMA (Cell Signaling Technology) at 4°C overnight. Then they were incubated with FITC anti-mouse IgG (Proteintech, Rosemont, IL, USA) for 1 h. After washing three times with PBA, slices were stained with diamidino-phenyl-indole (DAPI) to detect the nuclei. Images were captured using a laser scanning confocal microscope (Leica TCS SP5, Wetzlar, Germany).

### Hematoxylin-eosin (HE) staining

HE staining was used for the observation of vascular morphology. Briefly, sections of penis tissues were stained with hematoxylin solution and 0.5% eosin solution. After washing, the sections were differentiated with ethanol and xylene of different concentration gradients. After fixing the slice with neutral gum, images were captured by using Leica microscope (Carl Zeiss, Germany).

### Cell apoptosis analysis

Cell apoptosis was detected using a FITC Annexin V Apoptosis Detection Kit (BD Biosciences). 1 × 10^6^ cells were collected and incubated with Annexin V-FITC and propidium iodide (PI) for 15 min at 4°C. After that, cells were washed by pre-cooled PBS for 3 times and then suspended in buffer. The apoptosis rate was analyzed by flow cytometry.

### Statistical analysis

Data were represented as means ± standard deviation (SD) and analyzed for the significant difference using SPSS (Version 19.0; IBM, Armonk, NY, USA). Differences between two groups were tested by performing the *t*-test and between multiple groups by performing the ANOVA test. When *P* was less than 0.05, the difference was considered as statistically significant.

### Availability of data and materials

For data availability, please contact the corresponding author.

### Ethics approval and consent to participate

All experimental protocols were approved by the Committee of Animal Care and Use at the Third Affiliated Hospital of Guangzhou Medical University.

## RESULTS

### Establishment of miR-126 overexpression in MDSCs

**Figure 1:**
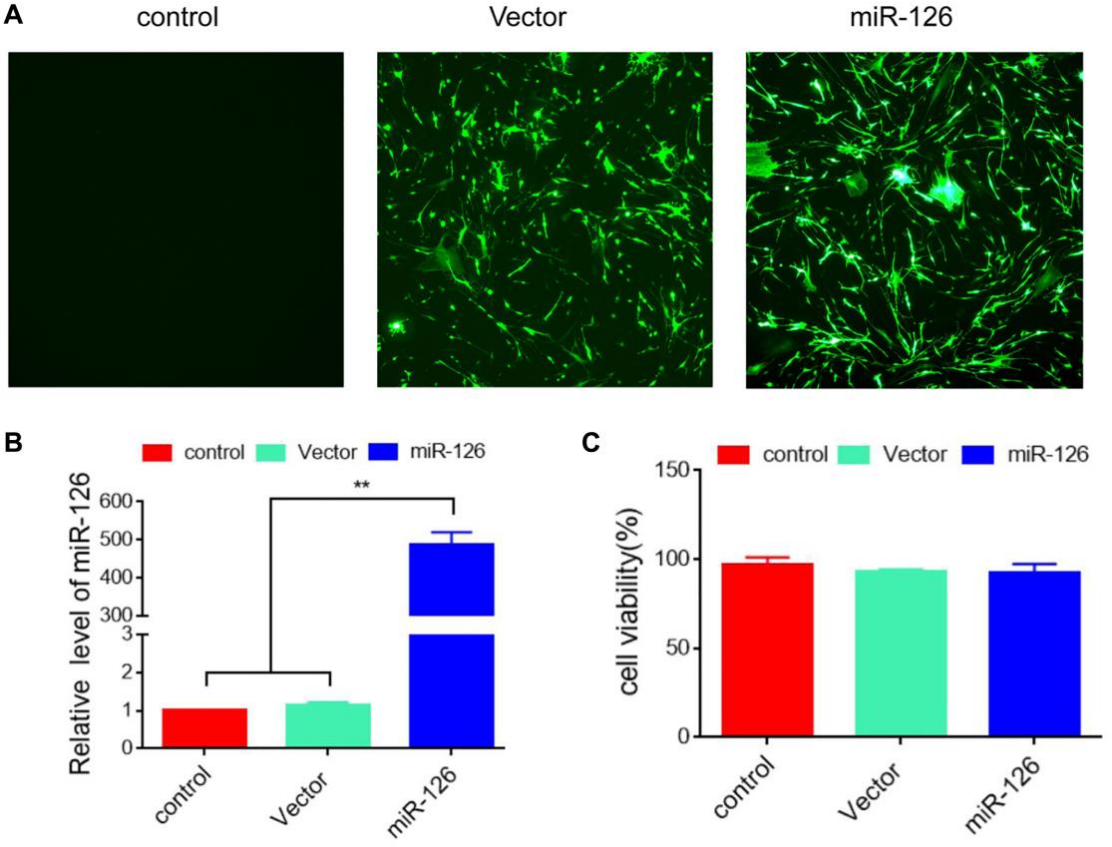
**Establishment of MDSCs overexpressing miRNA-126.** (**A**) 48 h after transfection, the efficiency was identified using immunofluorescence. (**B**) The expression level of miRNA-126. (**C**) CCK-8 assay for cell viability. Data are shown as the means ± SD. ^*^P < 0.05, ^**^P < 0.01.

MDSCs were transfected with lentivirus expressing miR-126/GFP, and transfection efficiency was identified by immunofluorescence staining (Figure 1A). Then, qRT-PCR analysis was performed and found that the level of miR-126 were elevated remarkably, indicating the successful establishment of miR-126 overexpression in MDSCs (Figure 1B). We detected whether miR-126 affected cell activity by CCK-8 assay, and found that the viability of MDSCs was not reduced by miR-126 transfection (Figure 1C).

### Enhancement of miR-126-overexpressing MDSCs in erectile response

**Figure 2:**
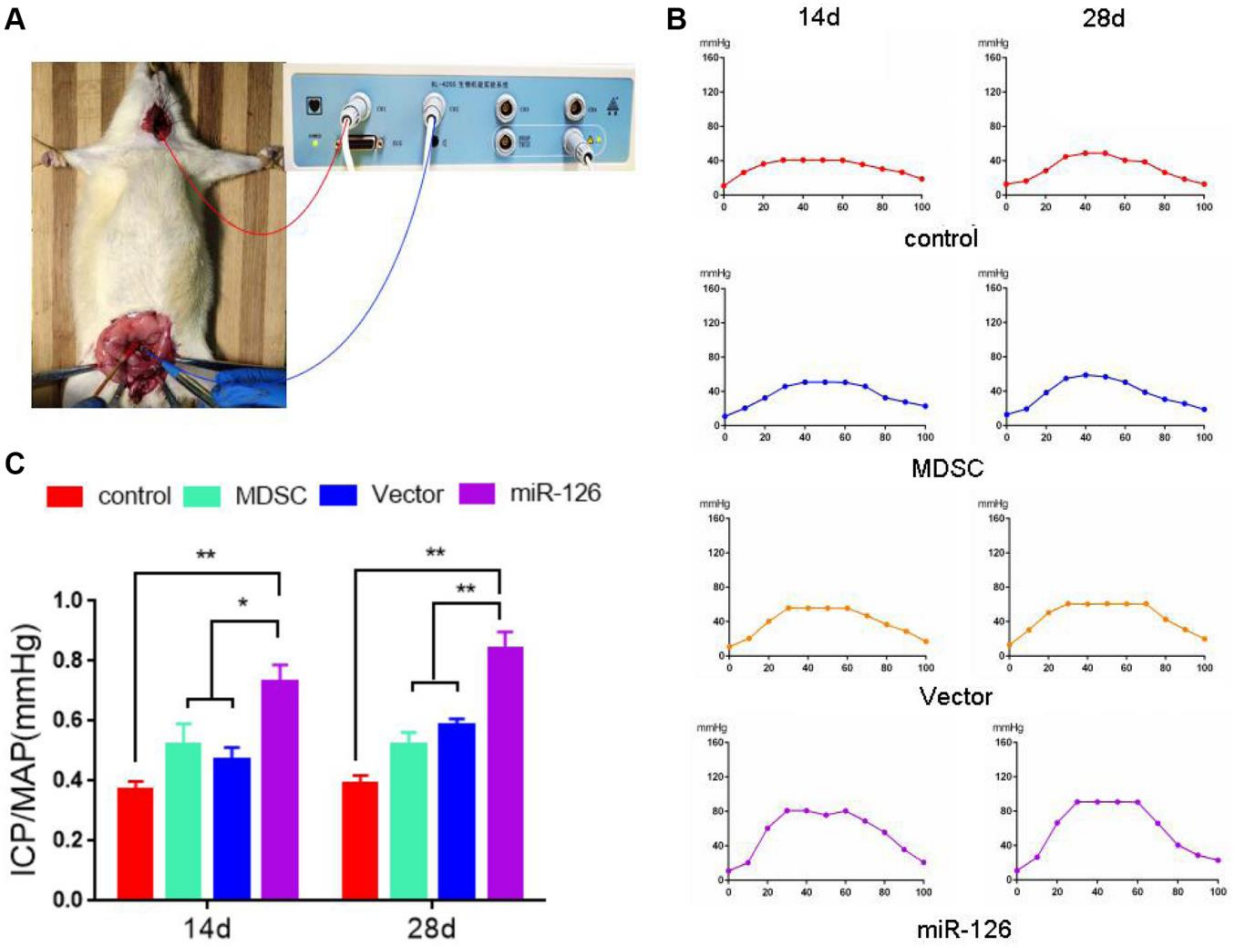
**The measurement of erectile response after injection with MDSCs.** (**A**) Images of operation. (**B**) Intra-cavernous pressure (ICP). (**C**) The value of ICP/ mean arterial pressure (MAP). Data are shown as the means ± SD. ^*^P < 0.05, ^**^P < 0.01.

Next, miR-126-overexpressing MDSCs were injected into rats for 14 days. The erectile function was observed by detecting the values of ICP/MAP, operations of which were shown in Figure 2A. The results of Figure 2B, 2C depicted that after injection for 14 days, the ICP/MAP values in the miR-126 group (0.75 ± 0.15) were notably higher than those in the Control group (0.37 ± 0.07), as well as MDSC and Vector groups (0.54 ± 0.16, 0.49 ± 0.12, respectively). After injection for 28 days, compared with the ICP/ MAP values in the Control (0.38 ± 0.07), MDSC (0.54 ± 0.11) and Vector groups (0.60 ± 0.02), the ICP/ MAP values in the miR-126 group (0.84 ± 0.14) increased significantly.

### Improvement of miR-126-overexpressing MDSCs in vascularization of the penis tissues

**Figure 3:**
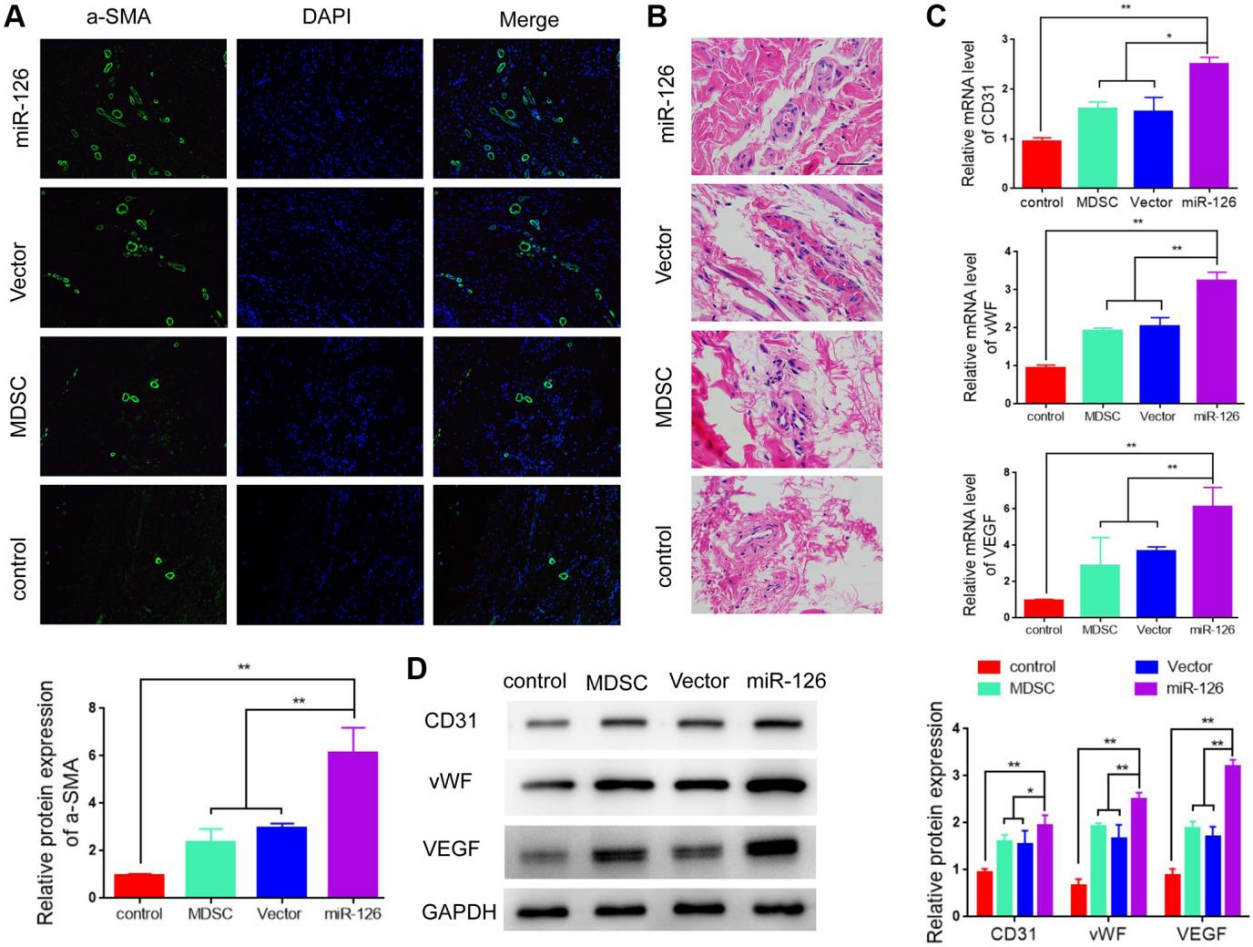
**miR-126-overexpressing MDSCs increases smooth muscle contents and improves vascular function in rats.** (**A**) Immunofluorescence images of α-SMA in the penis tissues. (**B**) H & E staining for morphologic detection. (**C**) mRNA levels of CD31, vWF and VEGF. (**D**) Protein expression of α-SMA, CD31, vWF and VEGF in rats. Data are shown as the means ± SD. ^*^P < 0.05, ^**^P < 0.01.

In order to further test the ability of miR-126 in enhancing revascularization, we observed the effect on increasing smooth muscle content by detecting the expression of α-SMA. The results in Figure 3A showed that rats injected with miR-126-overexpressing MDSCs had a higher α-SMA levels than rats in other three groups. At the same time, we also performed HE staining for the histological examination. The vascular structure was clearly more abundant in the miR-126 group than that in the other implanted groups (Figure 3B). Then we measured the expressions of CD31, von Willebrand Factor (vWF) and vascular endothelial factors (VEGF). The results in Figure 3C and 3D showed that the mRNA and protein expression of CD31, vWF and VEGF in the miR-126 group were remarkably higher than those of other three groups, indicating improved vascular function. In addition, TUNNEL staining was used for the detection of the degree of tissue necrosis, and showed that miR-126 significantly improved tissue necrosis (Supplementary Figure 1). All above findings demonstrated that the injection of miR-126-mediated MDSCs improved revascularization through increasing the content of SMCs and improving vascular function.

### Exosomes containing miR-126 promote angiogenesis and attenuate apoptosis in HUVECs

**Figure 4:**
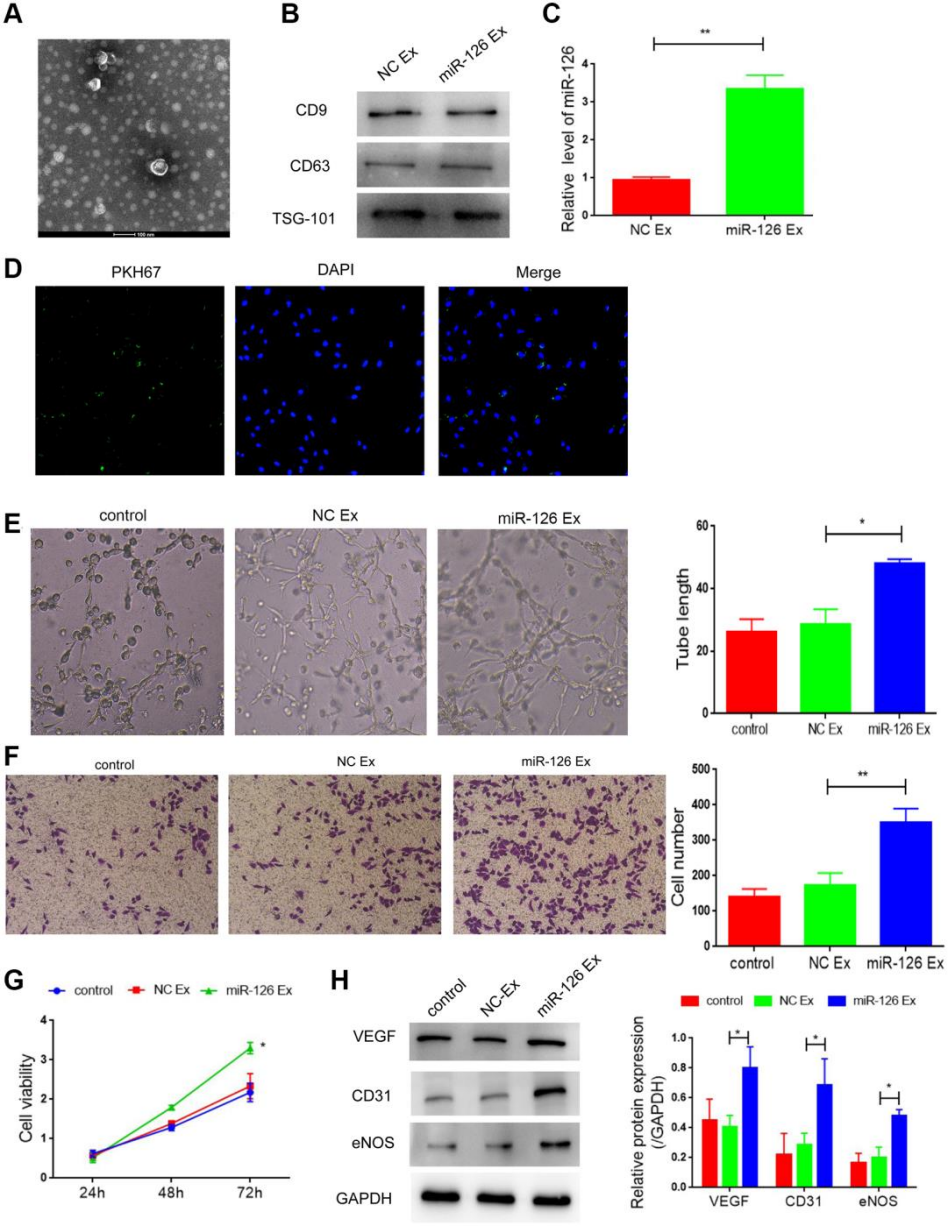
**Exosomes derived from miR-126-modifified MDSCs promote angiogenesis and attenuate apoptosis in HUVECs.** (**A**) Transmission electron photomicrograph of EXs. (**B**) Protein expression of CD9, CD63 and TSG-101. (**C**) mRNA-126 levels. (**D**) Confocal images of PKH67-labeled EXs taken up by HUVECs. (**E**) Tube formation was measured after seeding HUVECs pretreated with PBS, miR-con EXs or miR-126 EXs. Photomicrographs of tube-like structures and quantification of the tube number. (**F**) Representative microscopy images and quantitative analysis of apoptosis of HUVECs. (**G**) Cell viability. (**H**) Protein expression of α-SMA, CD31, vWF and VEGF in HUVECs. Data are shown as the means ± SD. ^*^P < 0.05, ^**^P < 0.01.

Previous studies suggested that the treatment effect based on miR-126 is limited due to the low efficiency of miRNA delivery *in vivo*. Recently, increasing evidence indicate that EXs might be used as a valuable therapeutic vehicle for miRNA delivery. Therefore, we explored the therapeutic effect of EXs containing miR-126 derived from MDSCs on cavernosa injury-induced erectile dysfunction. EXs were extracted from the supernatants of MSCs at 72 h after transfection of miR-126. TEM showed that EXs had a typical cup shape (Figure 4A). Then Western blotting was performed for the determination of the expression of EX markers (CD63, CD9 and TSG-101), which showed that these markers were highly expressed in both groups (Figure 4B). In addition, qRT-PCR was used for the measurement of miR-126 level in the EXs. Elevated miR-126 level was observed in EXs derived from MSCs transfected with the miR-126 mimic (Figure 4C). These results demonstrated that MDSCs efficiently transferred miR-126 into the secreted EXs.

Next, EXs extracted from MDSCs were labeled with PKH67 dye (green) and co-cultured with HUVECs. Figure 4D suggested that the labeled EXs were taken up by HUVECs. Then, we detected the effect of EXs containing miR-126 on angiogenesis under oxygen glucose deprivation. After treatment of EXs containing miR-126, the number of tube-like structures was markedly higher than that the other two groups, (Figure 4E, *P* < 0.05). Moreover, EXs containing miR-126 significantly inhibited apoptosis of HUVECs (Figure 4F and 4G; Supplementary Figure 2). All of above results indicated the role of EXs containing miR-126 in promoting angiogenesis. Additionally, miR-126 EXs treatment group also increased the protein expression of CD31, vWF and VEGF, indicating the improved vascular function (Figure 4H, all *P* < 0.05).

### Identification of insulin receptor substrate 1 (IRS1) and Krüppel-like factor (KLF10) as downstream targets of miR-126

**Figure 5:**
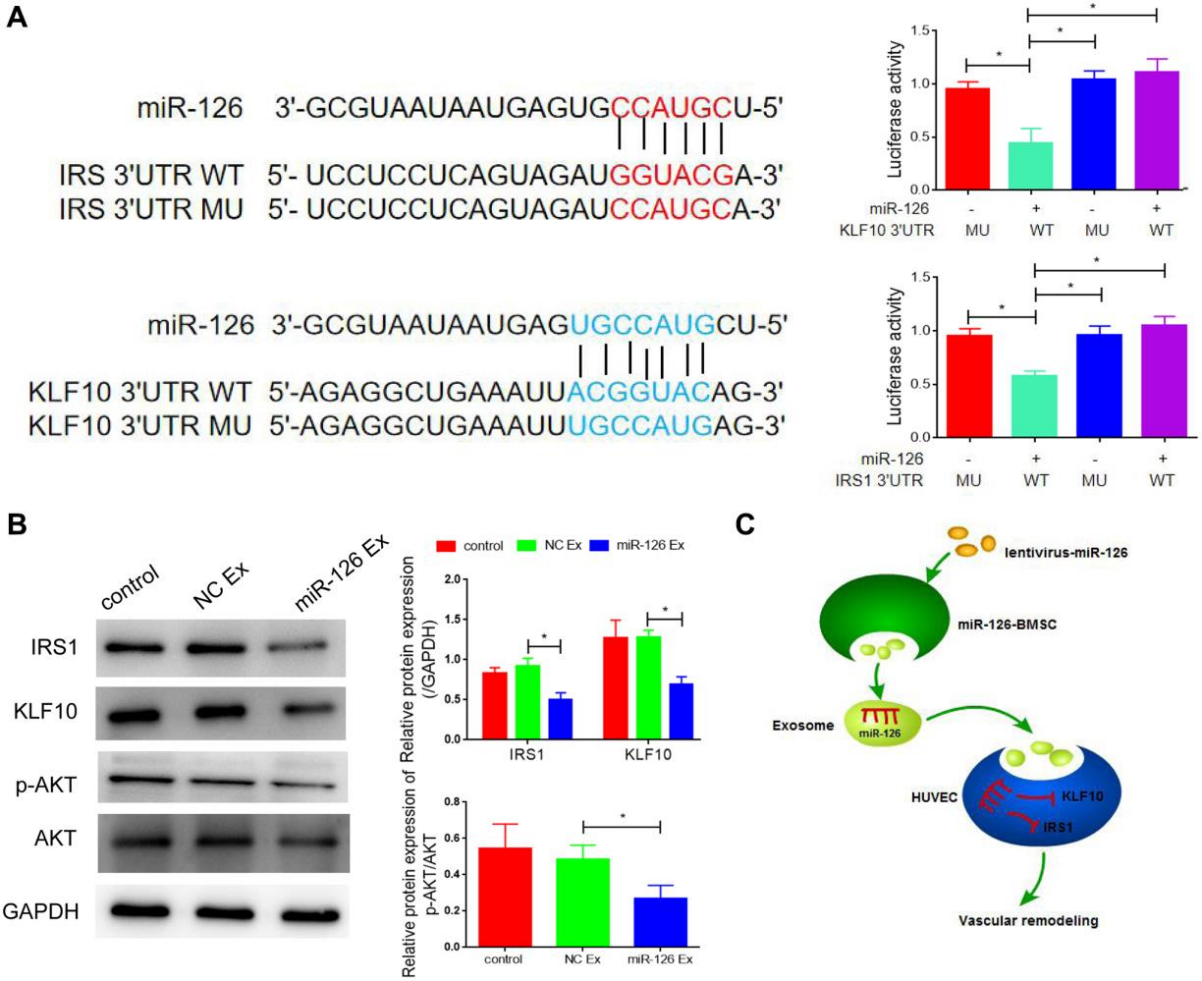
**IRS1 and KLF10 are targets of miRNA-126.** (**A**) The predicted binding sites between miRNA-126 and IRS1, and KLF10, and relative luciferase activity in IRS1 and KLF10 3′UTR (wild-type/mutant) and miRNA-126. (**B**) mRNA levels of IRS1 and KLF10 in MDSCs. (**C**) Protein expressions of IRS1 and KLF10 in MDSCs. Data are shown as the means ± SD. ^*^P < 0.05, ^**^P < 0.01.

miRNAs take their actions through the binding to downstream target genes. After the utilization of Targetscan and miRDB databases, IRS1 and KLF10 were predicted as the potential targets of miR-126, which was shown in Figure 5A. To identify the direct interaction between IRS1, KLF10 and miR-126, we performed the luciferase reporter assay. The results indicated that MDSCs transfected with miR-126 reduced the luciferase activity of the wild type IRS1 3′UTR and KLF10 3′UTR but did not decrease the activity of mutant types (Figure 5A). To further confirm results above, qRT-PCR analysis and Western blotting were carried out to determine the expression of IRS1and KLF10 in miR-126-overexpressing MDSCs. In miR-126-overexpressing MDSCs, the protein expression of IRS1 and KLF10 was reduced significantly (about by 1.9-folds and 2.2-folds, respectively) (Figure 5B). After transfection of IRS1 and KLF10 respectively, we detected the angiogenesis, and found that the improvement of miR-126 on angiogenesis was inhibited after overexpression of these two genes (Supplementary Figure 3). Taken together, IRS1 and KLF10 were confirmed target genes of miR-126. Lastly, miR-126 engineered MDSCs notably repaired cavernosa injury in rats via vascular reconstruction by exosomes (Figure 5C).

## DISCUSSION

In order to treat cavernosa injury-induced ED, investigators used various methods to reconstruct cavernous function, such as reconstruction of the penis by implanting prosthesis or cartilage, and achieved certain curative effects [17, 18]. However, due to its high technical difficulty and inexact effect, it has not been widely applied in clinical practice [18]. Therefore, drug therapy is still the main methods of ED prevention and treatment. PDE5 inhibitors are the most commonly used first-line treatment drugs, via improving endothelial function [4]. However, the pathogenesis of cavernosa injury-induced ED is caused by the interaction of nerves, vascular endothelium and smooth muscle [19]. The effective rate of PDE5 inhibitor was only 35% in patients with cavernosa injury-induced ED [19].

With the development of stem cells in the field of tissue repair and regenerative medicine engineering, a variety of stem cells have been used for the reconstruction of corpus cavernosum, such as bone marrow mesenchymal stem cells (BMSCs), ADSCs and urine-derived stem cells (UDSC) [20, 21]. Garcia et al. [22] found that the injection of ADSCs not only inhibited the apoptosis process of penile sponge tissue, but also promoted the retainment of endothelial cells, thus improving reconstructive corpus cavernosum of rats. Bharadwaj et al. [23] reported that UDSCs obtained from the upper urinary tract differentiated into urothelial and smooth muscle cells, and could serve as a potential stem cell source for bladder tissue engineering. Moreover, investigators suggested that stem cells combined with gene intervention could further enhance the therapeutic effect of ED. Messina et al. [24] transplanted mesenchymal stem cells (MSCs) and VEGF-modified-MSCs into rats. The results found that compared to MSCs-transplanted rats, VEGF-modified-MSCs-transplanted rats had higher cell viability and stronger ED function. The treatment of PTN-modified-ADSCs also showed better efficacy in the treatment of ED [25]. In the present study, we found that transplantation of micRNA-126-overexpressing MDSCs significantly attenuated cavernosa injury in rats, demonstrating an essential role of MDSCs combined with miR-126 in the treatment of ED.

MicroRNAs regulate tissue regeneration and stem cell differentiation *in vivo* [26]. They can stimulate the mechanism of intracellular repair or guide the differentiation of cells [26]. Therefore, the method of microRNAs transfected stem cells acting on tissue directed differentiation is expected to be used in the treatment of many diseases. For instance, Deng et al. [27] indicated that miR-31 transfected-BMSCs promoted the differentiation of osteogenic and repairment of bone defects. MiR-206 transfection enhanced the differentiation of MSCs into muscle cells, which had a positive effect on relieving muscle damage [28]. Stem cells modified by miRNA were also applied in ED therapy. Wang et al. [29] indicated that microRNA-125b promoted the differentiation of MSCs in ED rats by targeting BMPR1b. MicroRNA-320 has been reported to enhance the adipocytic differentiation of MSCs [30]. MicroRNA-145 modification promoted MSCs differentiate into vascular SMCs, resulting in the improvement of ED [31].

Exosomes are membrane vesicles released to the outside of cells after the fusion of intracellular vesicles and cell membrane, which act as the carrier of the intercellular signal transmission [32]. With the researches of functional proteins, mRNA and miRNA contained in exosomes, the role of exosomes in signal transduction has gradually been recognized. Accumulating studies have indicated that exosomes have a vital role in the diagnosis and treatment of cardiovascular diseases, tumors, and neurodegenerative diseases [33, 34]. Zeng et al. found that miR-25-3p can be transferred from colorectal cancer (CRC) cancer cells to vascular endothelial cells through exosomes, which promotes the metastasis of CRC [35]. Exosomes containing circRNAs secreted by adipocytes promote tumor growth and reduce DNA damage by inhibiting miR-34a and activating USP7/cyclin A2 signaling pathway [36]. In this study, we found that the exosomes secreted by MDSCs as the carrier of miR-126 was transported to the injured cavernous tissues, thus promoting vascular regeneration and inhibiting apoptosis.

Corpus cavernosum is a vascularized tissue composed of SMCs and ECs. Sufficient corpus cavernosum smooth muscle and sinus endothelium are the necessary condition for penile erection [37]. Therefore, increasing the number of SMCs and ECs were the key step for the improvement of ED. Li et al. [38] suggested that MDSCs possesses the ability to differentiate into SMCs and attenuate ED. Kovanecz et al. [39] also indicated the existence of MDSCs in the rat corpus cavernosum and MDSCs might be considered as a potential therapeutic treatment for ED in aging rats. Studies have also been reported that miR-126 is involved in smooth muscle growth, differentiation, and function. Zhou et al. [13] demonstrated that endothelial miR-126 increases the numbers of SMCs. Zhu et al. [15] indicated that miR-126 in ADSCs-derived exosomes enhances the contents of SMCs and ECs in diabetic rats. In this study, we found that miR-126 transfection enhanced vascular regeneration by promoting MDSCs differentiate into SMCS and ECs, and thus improving ED.

To further investigate the mechanism of miRNA-126-overexpressing MDSCs-mediated treatment of cavernosa injury, we identified the downstream target of miR-126. Both IRS1 and KLF10 were determined as direct target genes of miR-126 in our study. IRS1 is a multifunctional transcription factor, which has been proved to have important physiological functions in cytokine signal transduction and cell growth regulation [40]. Xi et al. reported that overexpression of IRS1 induced proliferation of vascular smooth muscle cells [41]. IRS1 exhibited an ability to inhibit the growth of coronary artery smooth muscle cells, thus suppressed neointimal hyperplasia [42]. KLF10 is a member of Krüppel -like transcription factor family [43]. Liu et al. [44] indicated that KLF10 was involved in early 3T3-L1 preadipocyte differentiation. In addition, investigators suggested that KLF10 modulated fibrosis of skeletal muscle [45]. In our study, IRS and KLF10 were confirmed target genes of miR-126, involved in the promotion of vascular reconstruction.

## CONCLUSION

Treatment of MDSCs transfected with miRNA-126 notably improved cavernosa injury in rats via promoting revascularization, increasing the content of SMCs and improving vascular function. We further demonstrated that miRNA-126-overexpressing MDSCs exerted this effect of vascular reconstruction by directly targeting IRS1 and KLF10.

## Supplementary Materials

Supplementary Figures

Supplementary Table 1
