# Correction for: MicroRNA-126 engineered muscle-derived stem cells attenuates cavernosa injury-induced erectile dysfunction in rats

**DOI:** 10.18632/aging.203704

**Published:** 2021-11-30

**Authors:** Zihao Zou, Muyuan Chai, Feixiang Guo, Xin Fu, Yu Lan, Shuqi Cao, Jianan Liu, Long Tian, Geng An

**Affiliations:** 1 Center for Reproductive Medicine, Key Laboratory for Major Obstetric Diseases of Guangdong Province, Third Affiliated Hospital of Guangzhou Medical University, Guangzhou, Guangdong, PR China; 2 National Engineering Research Center for Tissue Restoration and Reconstruction, South China University of Technology, Guangzhou, Guangdong, PR China; 3 Beijing Chao-Yang Hospital Capital Medical University, Beijing, PR, China

**Keywords:** correction

Original article: Aging. 2021; 13:14399–14415.  . https://doi.org/10.18632/aging.203057

**Copyright:** Zou et al. This is an open-access article distributed under the terms of the Creative Commons Attribution License (CC BY 3.0), which permits unrestricted use, distribution, and reproduction in any medium, provided the original author and source are credited.

**This article has been corrected:** The authors recently found error in the Figure 4F - the image for the NC-Ex group was incorrect. The authors corrected panel 4F in Figure 4 by using representative image from the original sets of experiments. This alteration does not affect the results or conclusions of this work. The authors would like to apologize for any inconvenience caused.

New Figure 4 is presented below.

**Figure 4:**
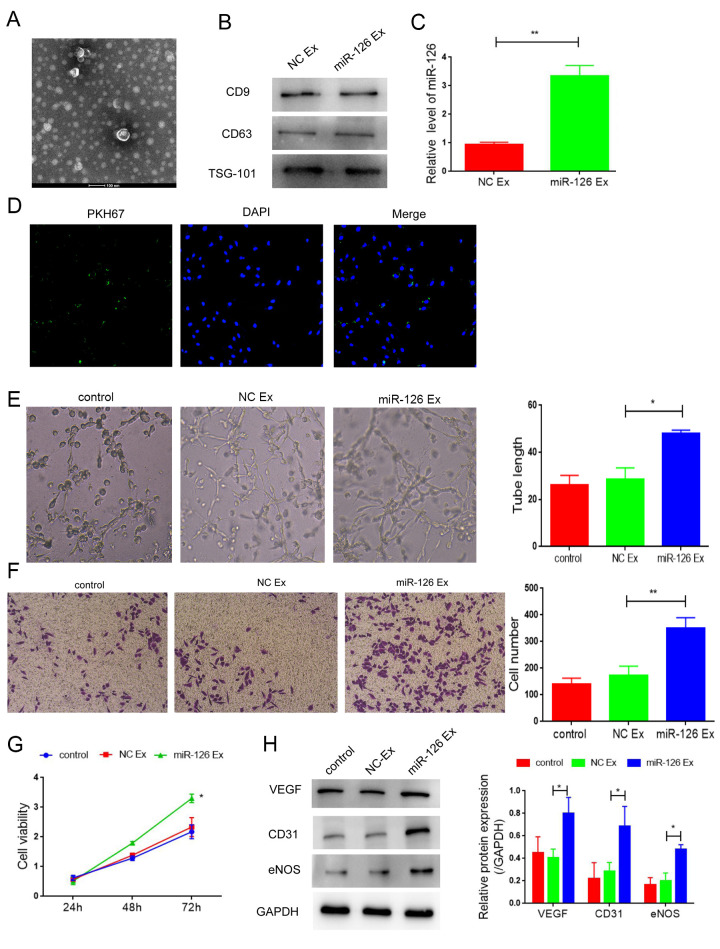
**Exosomes derived from miR-126-modifified MDSCs promote angiogenesis and attenuate apoptosis in HUVECs.** (**A**) Transmission electron photomicrograph of EXs. (**B**) Protein expression of CD9, CD63 and TSG-101. (**C**) mRNA-126 levels. (**D**) Confocal images of PKH67-labeled EXs taken up by HUVECs. (**E**) Tube formation was measured after seeding HUVECs pretreated with PBS, miR-con EXs or miR-126 EXs. Photomicrographs of tube-like structures and quantification of the tube number. (**F**) Representative microscopy images and quantitative analysis of apoptosis of HUVECs. (**G**) Cell viability. (**H**) Protein expression of α-SMA, CD31, vWF and VEGF in HUVECs. Data are shown as the means ± SD. ^*^P < 0.05, ^**^P < 0.01.

