# Supplementary material for: MicroRNA-126 engineered muscle-derived stem cells attenuates cavernosa injury-induced erectile dysfunction in rats
Source: Aging (Albany NY). 2021 May 23;13(10):14399–415. doi: 10.18632/aging.203057 (PMC8202866; doi:10.18632/aging.203057)
Supplement: Supplementary Figures [file aging-13-203057-s001.pdf]

SUPPLEMENTARY FIGURES

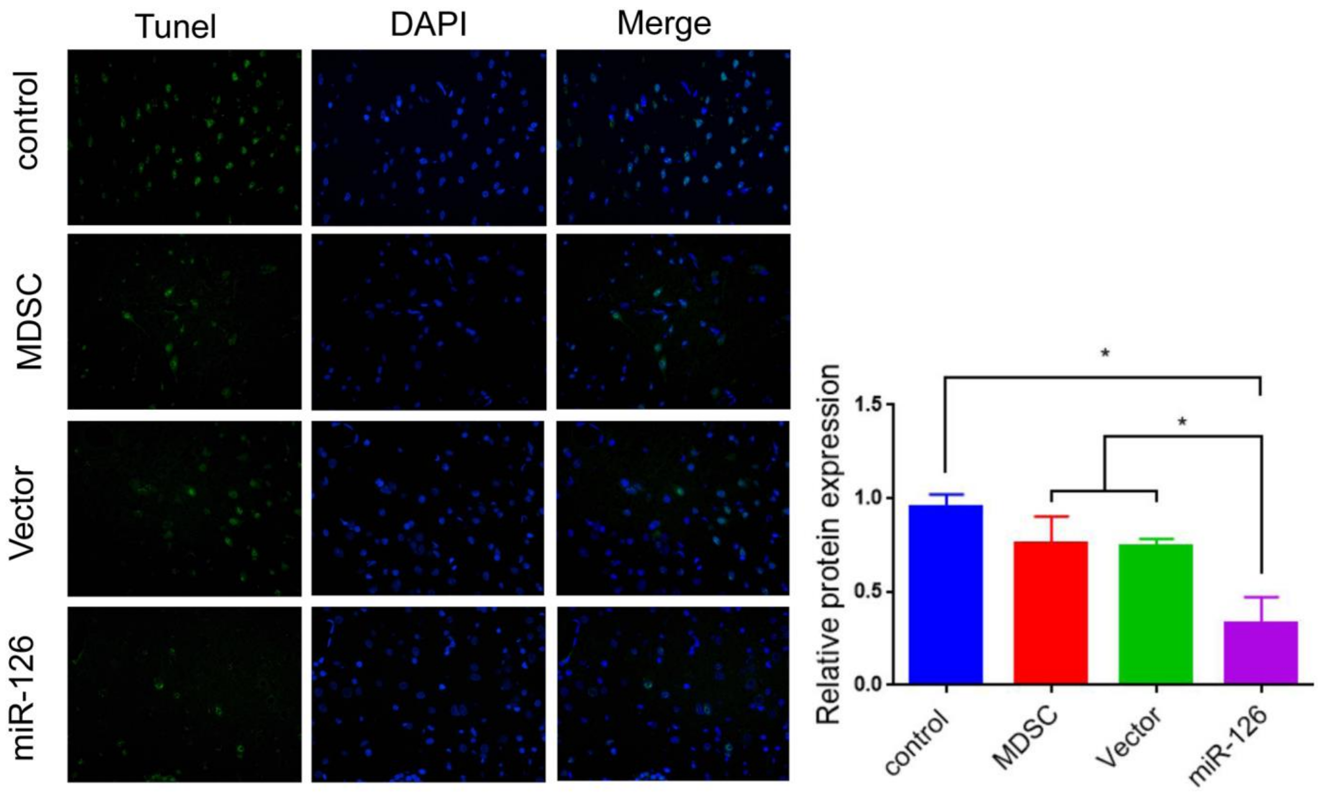

Supplementary Figure 1. miR-126-overexpressing MDSCs improved necrosis of penis tissue. TUNNEL staining for the detection of the degree of tissue necrosis. Data are shown as the means ± SD. $^{*}P < 0.05$, $^{**}P < 0.01$.

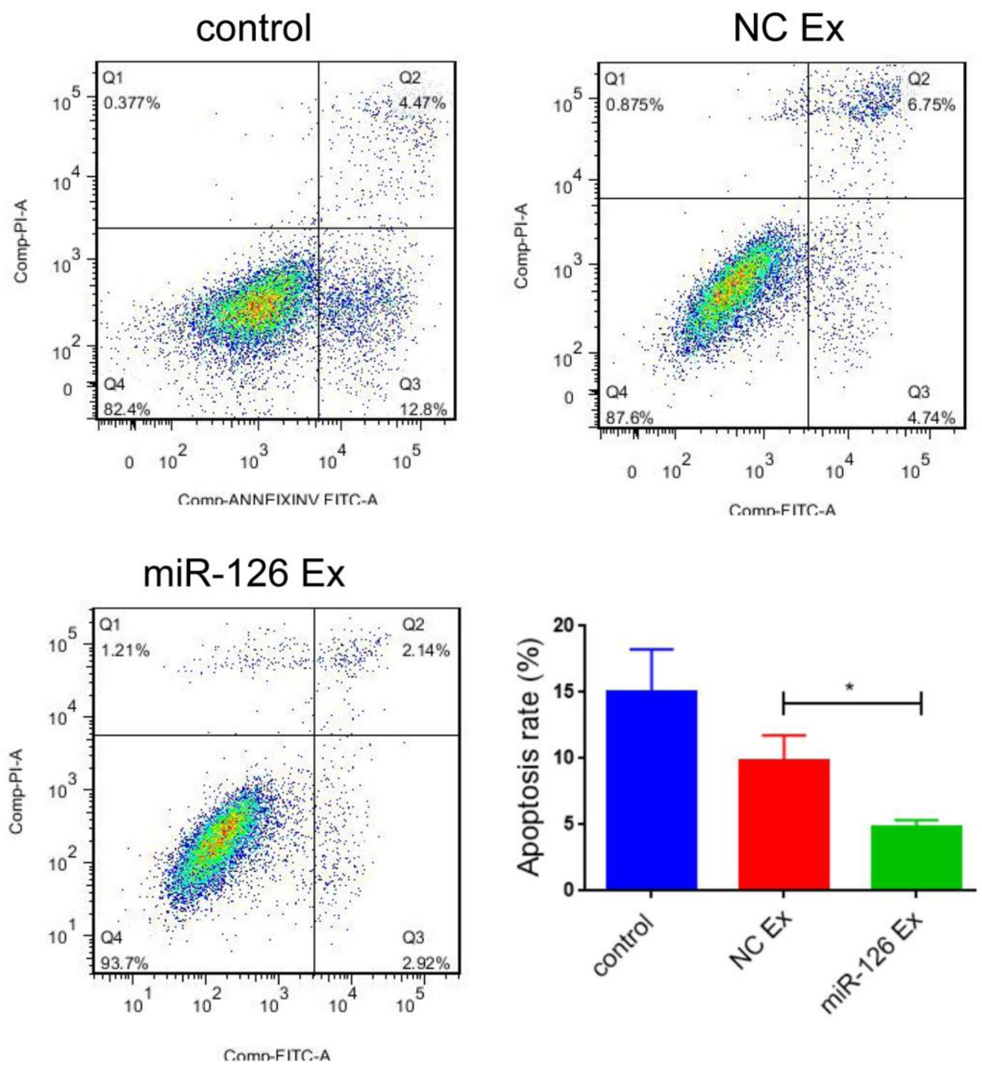

Supplementary Figure 2. Exosomes derived from miR-126-modified MDSCs inhibits apoptosis in HUVECs. The cell apoptosis rate by a FITC Annexin V apoptosis detection kit. Data are shown as the means ± SD. $^{*}P < 0.05$, $^{**}P < 0.01$.

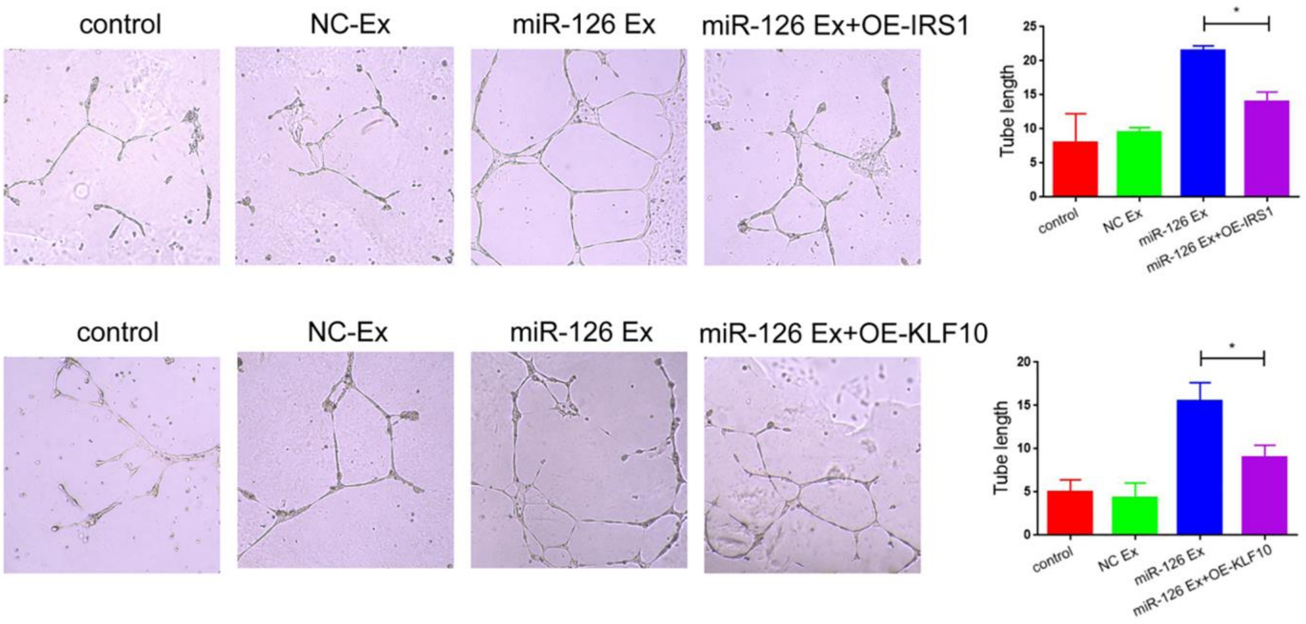

Supplementary Figure 3. Overexpression of IRS1 and KLF10 attenuates the improvement of miR-126 on angiogenesis. Photomicrographs of tube-like structures and quantification of the tube number. Data are shown as the means ± SD. $^{*}P < 0.05$, $^{**}P < 0.01$.
