# Supplementary material for: MicroRNA-126 engineered muscle-derived stem cells attenuates cavernosa injury-induced erectile dysfunction in rats
Source: Aging (Albany NY). 2021 May 23;13(10):14399–415. doi: 10.18632/aging.203057 (PMC8202866; doi:10.18632/aging.203057)
Supplement: Supplementary Table 1 [file aging-13-203057-s002.pdf]

SUPPLEMENTARY TABLE

Supplementary Table 1. Primer sequences used for qRT-PCR.

Genes	Primer Sequences (5′→3′)
MiR-126	F: AGCGATGATGCACTGTCAGAA
	R: AACGGAACTCCAGAAGACCAG
CD31	F: AACAGTGTTGACATGAAGAGCC
	R: TGTAAAACAGCACGTCATCCTT
vWF	F: GACTTTGAAGCCCCTGGACA
	R: GCCACCTCTCACTCCTAAGC
VEGF	F: GCACATAGAGAGAATGAGCTTCC
	R: CTCCGCTCTGAACAAGGCT
IRS1	F: ACAAACGCTTCTTCGTACTGC
	R: AGTCAGCCCGCTTGTTGATG
KLF10	F: CTTCCGGGAACACCTGATTTT
	R: GCAATGTGAGGTTTGGCAGTATC
β-actin	F: CGTAAAGACCTCTATGCCAACA
	R: CGGACTCATCGTACTCCTGCT
